# Deficiency of PKCλ/ι alleviates the liver pathologic impairment of *Schistosoma japonicum* infection by thwarting Th2 response

**DOI:** 10.1186/s13071-022-05283-x

**Published:** 2022-05-03

**Authors:** Congjin Mei, Yingying Yang, Panpan Dong, Lijun Song, Yonghua Zhou, Yongliang Xu, Chuanxin Yu

**Affiliations:** 1 National Health Commission Key Laboratory of Parasitic Disease Control and Prevention, Jiangsu Provincial Key Laboratory on Parasite and Vector Control Technology, Jiangsu Institute of Parasitic Diseases, 117 Meiyuan Yangxiang, Wuxi, 214064 Jiangsu China; 2 Public Health Research Center, Jiangnan University, Wuxi, 214122 Jiangsu China

**Keywords:** *Schistosoma japonicum*, PKCλ/ι, Th2 polarization, Granuloma, Liver fibrosis

## Abstract

**Background:**

The activation of immune response driven by the eggs of *Schistosoma japonicum* and the subsequent secretions is the culprit behind granulomatous inflammation and liver fibrosis. Evidence suggests that PKCλ/ι participates in a variety of physiological and pathological processes, including the regulation of metabolism, growth, proliferation and differentiation of cells. However, the role of PKCλ/ι in liver disease caused by *Schistosoma japonicum* remains unclear.

**Methods:**

In the present study, we observe the pathological changes of egg-induced granulomatous inflammation and fibrosis in the liver of mice infected by *Schistosoma japonicum* by using conditional PKCλ/ι-knockout mice and wild-type control. Immune cytokines and fibrogenic factors were analyzed by performing flow cytometry and real-time fluorescence quantitative PCR.

**Results:**

The results of H&E and Masson staining show that the degree of granulomatous lesions and fibrosis in the liver of the infected PKCλ/ι-knockout mice was significantly reduced compared with those of the infected wild-type mice. The mean area of single granuloma and hepatic fibrosis in the PKCλ/ι-knockout mice was significantly lower than that of the wild-type mice (85,295.10 ± 5399.30 μm^2^ vs. 1,433,702.04 ± 16,294.01 μm^2^, *P* < 0.001; 93,778.20 ± 8949.05 μm^2^ vs. 163,103.01 ± 11,103.20 μm^2^, *P* < 0.001), respectively. Serological analysis showed that the ALT content was significantly reduced in the infected knockout mice compared with infected wild-type mice. RT-PCR analysis showed that IL-4 content in knockout mice was significantly increased after *Schistosoma japonicum* infection, yet the increase was less than that in infected wild-type mice (*P* < 0.05). PKCλ/ι deficiency led to reduced expression of fibrosis-related factors, including TGF-β1, Col-1, Col-3, α-SMA and liver DAMP factor HMGB1. Flow cytometry analysis showed that the increasing percentage of Th2 cells, which mainly secrete IL-4 cytokines in spleen cells, was significantly lower in PKCλ/ι-deficient mice compared with wild-type mice after infection (*P* < 0.05).

**Conclusions:**

Our data demonstrate that PKCλ/ι deficiency alleviating granulomatous inflammation and fibrosis in the liver of mice with *S. japonicum* infection by downregulating Th2 immune response is the potential molecular mechanism behind the role of PKCλ/ι in schistosomiasis.

**Graphical Abstract:**

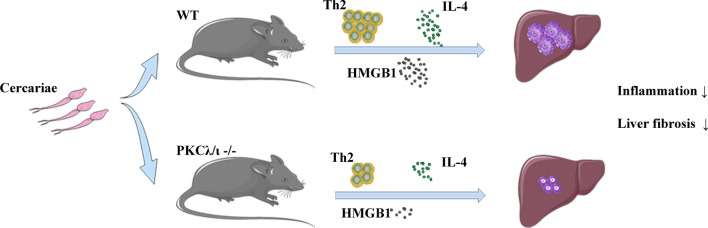

## Background

Schistosomiasis is a zoonosis widely prevalent in tropical and subtropical areas that severely damages human health. Approximately 760 million people are dwelling in schistosomiasis-endemic areas worldwide, and nearly 200 million people have been infected, raising the death toll to 200,000 on an annual basis [1]. The prevalence of schistosomiasis has seriously impeded social stability and economic prosperity in the epidemic areas. Three main species cause human schistosomiasis: *Schistosoma japonicum* (*S. japonicum*), *Schistosoma mansoni* (*S. mansoni*) and *Schistosoma haematobium* (*S. haematobium*) [2]. *Schistosoma japonicum* is an epidemic in China, especially in the areas along the Yangtze River. The adult schistosomes reside in the mesenteric vein of the host and produce eggs that enter the liver by hepatic portal vein blood flow. The schistosome eggs stay in the liver for a long time, and their secreted excreta wreck the surrounding liver tissue, stimulating an immediate response of the immune system, leading to granulomatous inflammation and even liver fibrosis, causing severe damage to the host [3]. Despite efforts to enhance our understanding, the exact pathogenesis of granuloma formation and fibrosis caused by *S. japonicum* continues to elude the medical community [4, 5].

Widely distributed in tissues and cells, protein kinase C (PKC) plays a key role in mediating transmembrane signaling. PKC can be divided into three groups: classical PKC (including α, βI, βII and γ subgroups), novel PKC (including δ, ε, η and θ subclasses) and atypical PKC (consisting of PKCζ and PKCλ/ι subclasses) [6]. PKC is composed of a single peptide chain, with its N-terminal being the regulatory region and C-terminal being the catalytic region. PKC regulates the metabolism, growth, proliferation and differentiation of a variety of cells by catalyzing the phosphorylation of Ser/Thr in proteins. PKC is involved in many physiological and pathological reactions and is implicated in tumor immunity, allergic inflammation, diabetes and other pathogeneses. PKC has now become a promising target for new drug development. It has been reported that atypical protein kinase C (PKCλ/ι) is a crucial signaling transduction molecule functioning to regulate and maintain Th1/Th2 homeostasis. The level of IL-4, a Th2 cell cytokine, was downregulated in PKCλ/ι-knockout mice, while the level of IFN-γ, a Th1cell cytokine, remained roughly unaltered. The concept that PKCλ/ι deficiency can effectively ameliorate allergic bronchial asthma induced by ovalbumin (OVA) shows that PKCλ/ι is a key factor in downregulating Th2-skewed immune response [7].

Th1 immune response plays a pivotal role in early schistosome infection and can be transformed into Th2 immune response with time [8]. As schistosome adults begin to lay eggs, mostly 3 weeks after infection, Th2 immune response becomes dominant in the immune system. The overactive Th2 response can contribute to allergies and a variety of syndromes and even trigger autoimmune disease [9]. Many studies have demonstrated that cytokines secreted by Th2 immune cells act to activate and transform hepatic stellate cells (HSC) into fibroblast cells, stimulating the production of a large amount of collagen matrix and in turn causing inflammatory granuloma and liver fibrosis [10]. Therefore, Th2 immune response induced by schistosome egg antigen constitutes a potential mechanism of liver fibrosis. Hence, hindering Th2 immune response induced by schistosomes should be able to inhibit the egg granulomatous inflammatory response and suppress hepatic fibrosis, thereby alleviating pathological liver damage. To our knowledge, the potential effect of the knockout of PKCλ/ι on moderating liver damage caused by schistosome infection has not been investigated.

Herein, we established a model of *S. japonicum* infection using C57BL/6 J mice with conditional knockout of PKCλ/ι gene in an effort to explore the effects of PKCλ/ι deficiency on pathological liver damage and fibrosis induced by *S. japonicum* and to clarify the underlying molecular mechanism.

Our result showed that knockout of PKCλ/ι can effectively reduce the formation of liver granuloma, limit the extent of fibrosis in mice infected by *S. japonicum* and downregulate Th2 immune response. The current study sheds novel light on the key role of PKCλ/ι in regulating immune response and the pathological process of liver fibrosis in the murine host of schistosomiasis.

## Methods

### Experimental materials

#### Animals

Conditional PKCλ/ι knockout mice (PKCλ/ι^flx/flx^Cre^OX40^) were developed and maintained at the Model Animal Center of Nanjing University [11]. Our research team conducted the breeding, feeding and genotyping of the mice and then selected the eligible conditional PKCλ/ι knockout mice for the subsequent experiments. All animals were kept in an SPF environment and granted access to food and water.

The design and conduct of the study strictly follow the Guidelines for the Care and Use of Laboratory Animals of the Ministry of Science and Technology, PRC ([2006] no. 398). All experimental procedures were performed in accordance with the General Requirements for Laboratory Biosafety of the People's Republic of China (GB19489-2008), and the ethical approval of Jiangsu Institute of Parasitic Diseases has been obtained (approval no. [2020] 12). Every effort was made to minimize the suffering of the animals.

#### *Oncomelania hupensis* infected with *S. japonicum* and cercariae

*Oncomelania hupensis* infected with *S. japonicum* were provided by the snail room at Jiangsu Institute of Parasitic Diseases. Cercariae were hatched in the infected *Oncomelania hupensis* under incandescence in dechlorinated water at 20–25 ℃ for 2 h.

### Experimental method

#### Establishment of a schistosome infection model

Twenty C57BL/6 J mice (6–8 weeks old) and conditional PKCλ/ι knockout mice were selected for the experiments. The animals were randomly divided into four groups: WT group (WT group, genotype PKCλ/ι^flx/flx^, *n* = 5); WT group infected with *S. japonicum* (WTSJ group, genotype PKCλ/ι^flx/flx^, *n* = 5); KO group (KO group, genotype PKCλ/ι^flx/flx^Cre^OX40^, *n* = 5); KO group infected with *S. japonicum* (KOSJ group, genotype PKCλ/ι^flx/flx^Cre^OX40^, *n* = 5). Each mouse in the WTSJ group and KOSJ group was infected with 15 *S. japonicum* cercariae on the abdomen. At 7 weeks post-infection, the mice were anesthetized using CO_2_, and blood was collected through the eyeball. Serum was separated from the blood and stored at – 80 ℃. The intestines of the mice were removed and soaked in sterilized sodium citrate solution. The adult schistosomes in each mouse were counted after the schistosomes escaped from the mesenteric vessels. The liver was removed, weighed and digested overnight in 4% potassium hydroxide solution at 37 ℃. The eggs per gram of liver tissue were counted under an inverted microscope.

#### Determination of ALT and HMGB1 in serum

The content of alanine aminotransferase (ALT) in the serum was determined using a commercial alanine aminotransferase kit (cat. no. C009-2-1) produced by Nanjing Jiancheng Bioengineering Institute. The content of high mobility group protein B1 (HMGB1) in serum was detected by using HMGB1 detection kit for mice (no. E-El-M0676C) purchased from Elabscience Biotechnology. The procedures were carried out following the manufacturers' instructions.

#### H&E and Masson staining and determination of HYP in liver

The liver tissues were embedded and sectioned for H&E and Masson staining [12] using staining kits purchased from Beijing Solarbio Science & Technology Co., Ltd. (catalog no. G1120, G1346, respectively).

Films of H&E and Masson staining were read under an Olympus BX51 microscope to assess the egg granulomatous lesions and hepatic fibrosis in each group. Twenty-five single egg granulomas with clear and well-separated edges were randomly selected from each group. The average areas of the single granuloma and fibrosis were calculated using Cellsens Dimension software.

The content of hydroxyproline (HYP) in the liver tissues of mice was determined following the HYP kit (no. A030-2-1, Nanjing Jiancheng Bioengineering Institute) manufacturer's instructions.

#### Flow cytometry analysis

Spleen cells were isolated by using the physical grinding method. After the spleen cells had been stimulated by PMA and ionomycin, intracellular cytokines were stained to detect the expression levels of IFN-γ and IL-4, thereby investigating immune polarization of CD4^+^ T in the spleen, according to the method detailed in the previous study [11].

#### Real-time quantitative PCR (RT-PCR)

Total RNA from liver tissue was extracted using Trizol. RNA was reverse transcribed to cDNA using a reverse transcription kit (Roche, Basel, Switzerland), which was used as the template for real-time quantitative fluorescence PCR (RT-PCR). SYBR Green I Master (Roche, Basel, Switzerland) and LightCycler 480 System (Roche, Basel, Switzerland) were used to measure the mRNA expression levels of immune cytokines and fibrosis-related factors in the liver of each group. The primer sequences of cytokines and the fibrosis-related cytokines are listed in Table 1.

**Table 1:** Primer sequences of cytokines and fibrosis related factors

Cytokines	Primers	Sequence
18S	Forward	5ʹ-TGCACCACCAACTGCTTAGC-3ʹ
	Reverse	5ʹ-GTGGTCATGAGCCCTTCCA-3ʹ
IFN-γ	Forward	5ʹ-GATGCATTCATGAGTATTGCCAAGT-3ʹ
	Reverse	5ʹ-GTGGACCACTCGGATGAGCTC-3ʹ
IL-4	Forward	5ʹ-AGATCATCGGCATTTTGAACG-3ʹ
	Reverse	5ʹ-TTTGGCACATCCATCTCCG-3ʹ
TGF-β1	Forward	5ʹ-CTTTAGGAAGGACCTGGGTT-3ʹ
	Reverse	5ʹ-CAGGAGCGCACAATCATGTT-3ʹ
Col-1	Forward	5ʹ-GCGAGTGCTGTGCTTTCTG-3ʹ
	Reverse	5ʹ-TACCTCGACTCCTACATCTTC-3ʹ
Col-3	Forward	5ʹ-CCCAACCCAGAGATCCCATT-3ʹ
	Reverse	5ʹ-GAAGCACAGGAGCAGGTGTAGA-3ʹ
HMGB1	Forward	5ʹ-CGGATGCTTCTGTCAACTTCTC-3ʹ
	Reverse	5ʹ-GTTTCTTCGCAACATCACCAAT-3ʹ
IL-13	Forward	5ʹ-GCCAGCCCACAGTTCTACAGC-3ʹ
	Reverse	5ʹ-GTGATGTTGCTCAGCTCCTCA-3ʹ

#### Western blot

Total protein in liver tissue was extracted with RIPA lysis buffer (1 × PBS, 1% Nonidet P-40, 0.5% sodium deoxycholate, 0.1% SDS, 1 mM phenyl methyl sulfonyl fluoride and protease inhibitors). Equal protein (80 μg) was analyzed by SDS-PAGE electrophoresis, transferred onto a polyvinylidene fuoride (PVDF) membrane and then incubated with the antibodies against α-SMA or collagen 1 (Abcam). Protein bands were detected using the Bio-Rad ChemiDoc™ (Hercules, CA, USA). GAPDH was used as an internal control.

#### Cytokine assay

Spleen cells were activated with plate-bound anti-CD3 (5 µg/ml) plus soluble anti-CD28 (2 µg/ml, BD Bioscience) and stimulated with SEA (50 µg/ml) for 48 h. Cytokines in the supernatants were assayed by ELISA. IL-4 was assayed with OptEIA kits (BD PharMingen). ELISA plates were developed with TMB substrate (BD PharMingen) and read by a microplate reader (model 570, Bio-Rad).

#### Statistical analysis

Unless otherwise noted, the data in this article are presented as mean ± standard error of mean (SEM). *T*-test, one-way ANOVA, Mann-Whitney *U* test and Kruskal-Wallis test were used for statistical analysis by applying GraphPad Prism 5. *P* < 0.05 was considered to indicate statistical significance.

## Results

### PKCλ/ι deficiency could not alter the amounts of adult schistosomes and schistosome eggs in the infected mice

**Fig. 1:**
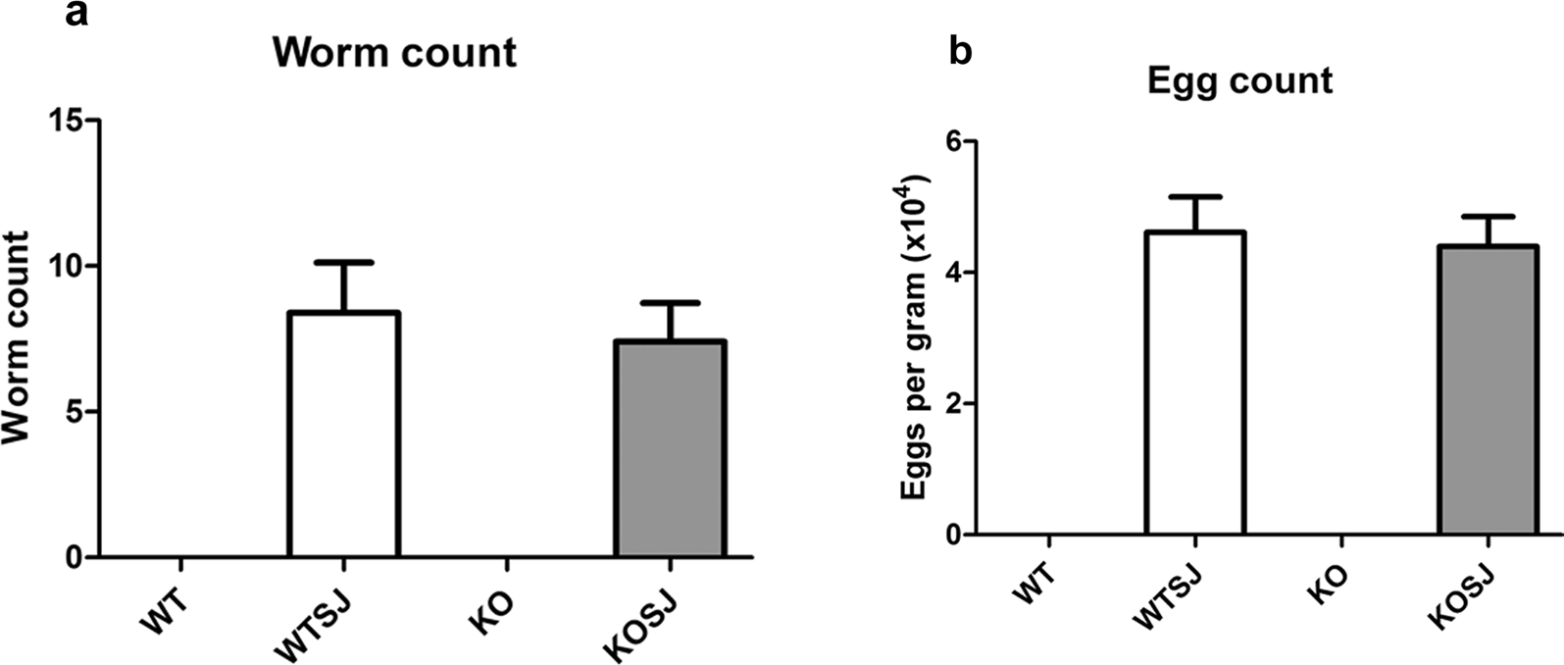
Comparison of worm loads and egg loads between WTSJ and KOSJ group. **a** The average number of worms did not significantly vary between the WTSJ and KOSJ group. **b** Average number of eggs per gram was not significantly different in the liver tissues of WTSJ and KOSJ group

The mice were dissected 7 weeks after infection; the number of adult schistosomes and eggs in the liver tissue were counted. The results showed that the average worm burdens in the WTSJ and KOSJ groups were 8.40 ± 1.70 and 7.40 ± 1.30 per mouse, respectively (Fig. 1a); hence, no statistical significance was observed (*P* > 0.05). The number of egg burdens in the liver in WTSJ and KOSJ groups was 46,117.00 ± 5399.00 vs. 43,995.00 ± 4506.00 eggs per gram, respectively (Fig. 1b), indicating no statistically significant difference (*P* > 0.05).

### PKCλ/ι deficiency reduces serum levels of ALT and HMGB1 in mice infected with *S. japonicum*

**Fig. 2:**
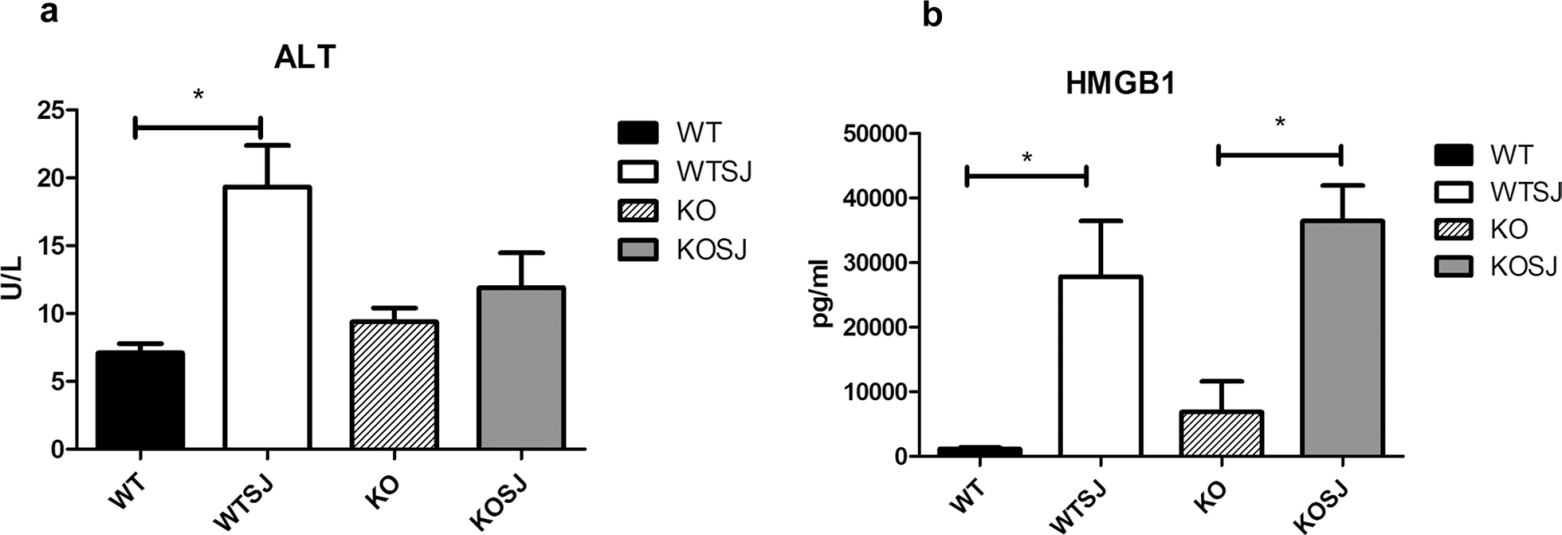
Content of ALT and HMGB1 in each group post-infection. **a** ALT level in each group after schistosome infection; **b** HMGB1 level in each group after schistosome infection (*P < 0.05; **P < 0.01; ***P < 0.001)

HMGB1 level in the serum of the murine host was determined by conducting ELISA; the serum levels of ALT were determined using the microplate method. The results showed that the ALT level in the WT group, WTSJ group, KO group and KOSJ group was 7.10 ± 0.70, 19.32 ± 3.10, 9.35 ± 1.00, and 11.90 ± 2.50 U/l, respectively (Fig. 2a). *Schistosoma japonicum* infection obviously elevated the content of ALT in serum in WTSJ and KOSJ mice, yet the increasing rate was lower in KOSJ group than that in WTSJ group. HMGB1 in WT group and WTSJ group was 1164.00 ± 201.00 and 27,820.00 ± 8612.00 pg/ml, respectively, indicating a 23.9-fold increase after infection. Consistently, HMGB1 concentrations showed a 5.3-fold increase in the KOSJ group compared with that in the KO group (36,441 ± 5491 vs. 6858 ± 4749 pg/ml). As illustrated in Fig. 2b, PKCλ/ι deficiency obviously suppressed the increasing proportion of serum HMGB1 levels induced by schistosome infection.

### PKCλ/ι deficiency alleviates liver granuloma and fibrosis in mice infected with *S. japonicum*

**Fig. 3:**
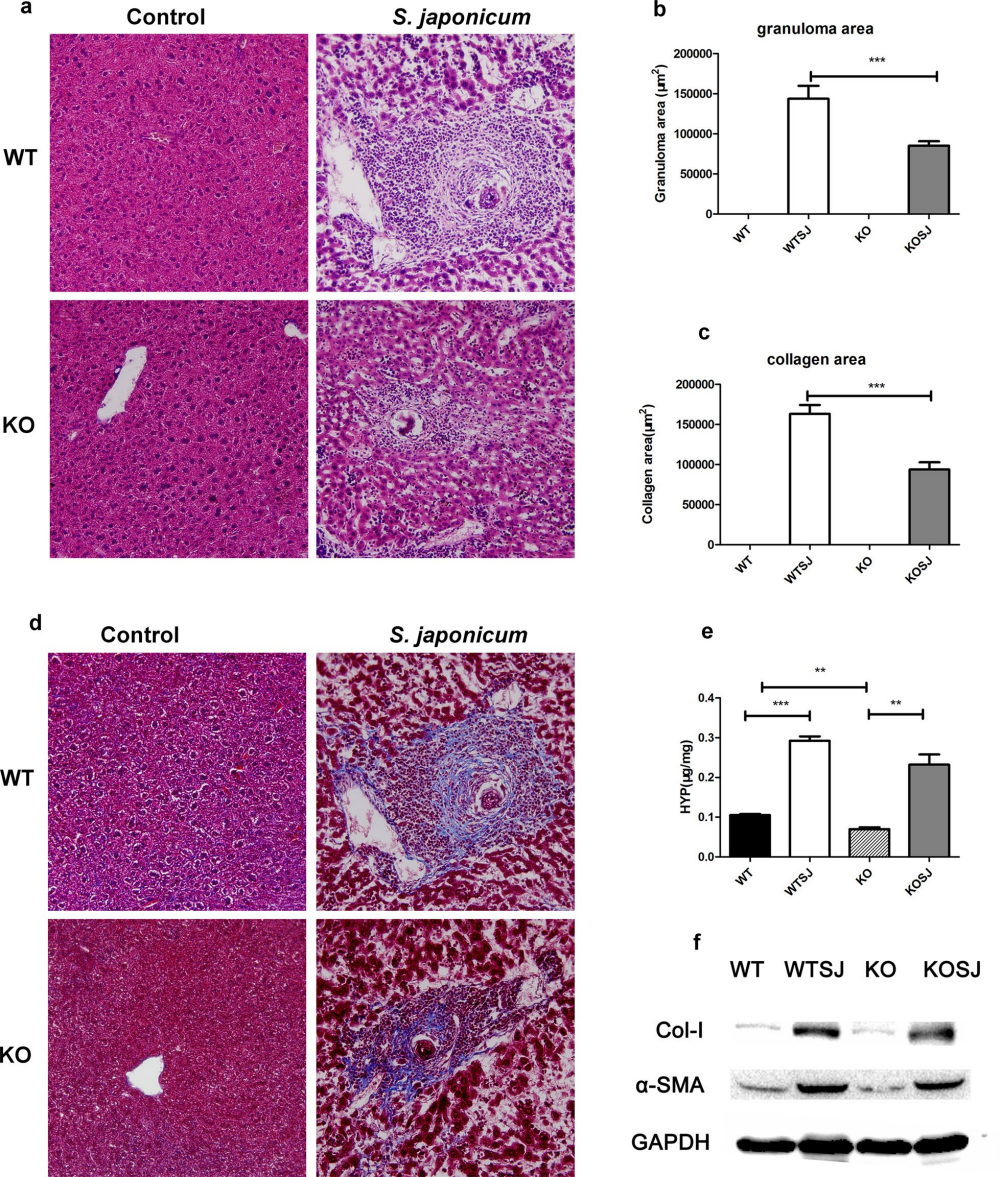
Pathological changes of liver tissue after schistosome infection. **a** H&E staining of pathological sections of liver tissue. **b** Statistical analysis results of average single granuloma in each group. **c** Statistical results of average collagen area of single granuloma by Masson staining. **d** Masson staining of pathological sections of liver tissue in each group. **e** Content of HYP in the liver tissue of each group. **f** Immunoblot of α-SMA and Col-1 expression in liver tissues (*P < 0.05; **P < 0.01; ***P < 0.001)

Seven weeks after infection, the mice showed obvious formation of egg granuloma in the liver and indication of hepatic fibrosis (Fig. 3a, d). The average area of single liver granuloma in the WTSJ was significantly higher than that in KOSJ group (1,433,702.04 ± 16,294.01 vs. 85,295.10 ± 5399.30 μm^2^, *P* < 0.001) (Fig. 3b). The area of single egg granuloma collagen in WTSJ group was significantly higher than that in KOSJ group (163,103.01 ± 11,103.20 vs. 93,778.20 ± 8949.05 μm^2^, *P* < 0.001) (Fig. 3c). These results suggest that PKCλ/ι loss-of-function mutation can significantly suppress egg granuloma and diminish fibrotic lesions.

As seen in Fig. 3e, the content of HYP was 0.11 μg/mg, 0.29 μg/mg, 0.07 μg/mg and 0.23 μg/mg, respectively. KO group exhibited significantly lower levels of HYP than WT group (*P* < 0.01). The content of HYP was significantly higher in WTSJ group compared to that in WT group (*P* < 0.001). Consistently, the content of HYP was significantly enhanced in KOSJ group compared to that in KO group (*P* < 0.01).

In addition, we examined the protein expression of α-SMA and Col-1 in liver tissue by immunoblotting. The results showed that α-SMA and Col-1 expression was reduced in KOSJ compared with that in WTSJ group (Fig. 3f).

### PKCλ/ι deficiency thwarts Th2 immune response and inhibits the secretion of hepatic fibrosis-related factors in liver of mice with *S. japonicum* infection

**Fig. 4:**
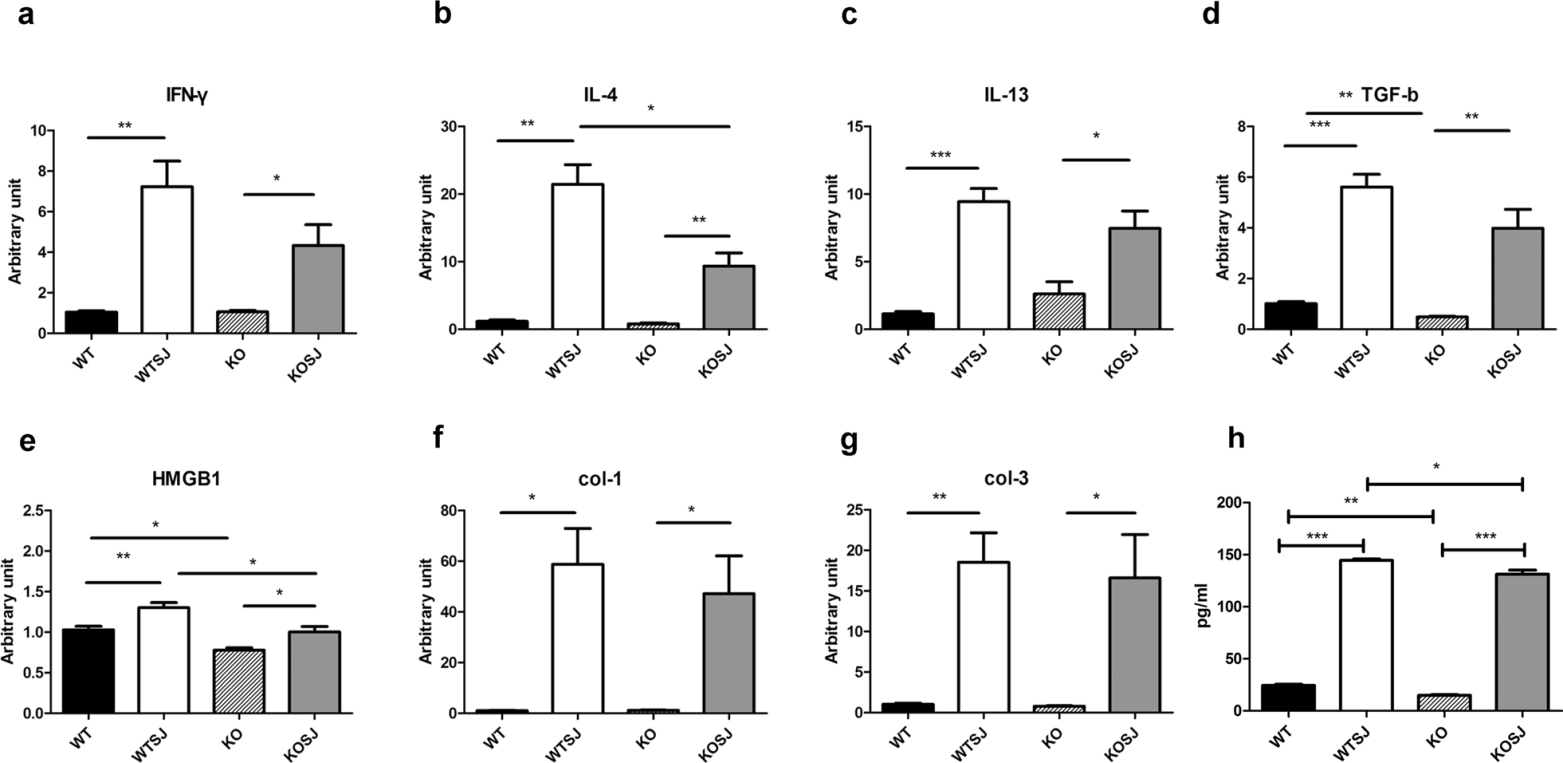
Changes in mRNA levels of liver immune cytokines and fibrosis-related factors in each group after schistosome infection. **a** mRNA relative expression level of IFN-γ. **b** Relative expression of IL-4 mRNA. **c** Relative expression level of IL-13 mRNA. **d** mRNA relative expression level of TGF-β1. **e** mRNA relative expression level of HMGB1. **f** mRNA relative expression level of Col-1. **g** Relative mRNA expression of Col-3. **h** IL-4 in the culture supernatants was assayed by ELISA (*P < 0.05; **P < 0.01; ***P < 0.001)

Compared with WT group, the level of IFN-γ was not obviously reduced in KO group. Although IFN-γ level in KOSJ group decreased as opposed to WTSJ group, the magnitude of the decrease did not reach statistical significance (Fig. 4a).

IL-4, which functions to induce the characteristic Th2 immune response, was significantly increased in both WTSJ group and KOSJ group after infection in mRNA and cytokine level (Fig. 4b, h). Of note, the increasing magnitude of IL-4 in the KOSJ group was significantly lower than that in the WTSJ group (*P* < 0.05). These results collectively indicate that schistosome infection acts to polarize immune response and facilitate the shift toward Th2-type response, with or without PKCλ/ι loss of function (KO group or WT group). Intriguingly, the conditional loss of PKCλ/ι gene effectively diminished Th2 polarization induced by schistosome infection.

The expression levels of fibrosis-related factors, such as TGF-β1 (Fig. 4d), Col-1(Fig. 4f) and Col-3 (Fig. 4g), in KOSJ group were notably lower than those in WTSJ group (3.98 vs. 5.61; 47.17 vs. 58.77; 16.59 vs. 18.54, respectively). Intriguingly, the levels of these fibrosis-related factors decreased after PKCλ/ι loss of function. The level of HMGB1, which signifies the extent of cell necrosis, in the KOSJ group was significantly lower than that in the WTSJ group (1.30 vs. 1.00, *P* < 0.05) (Fig. 4e).

### PKCλ/ι deficiency inhibits the polarization of CD4^+^T cells to Th2 induced by *S. japonicum*

**Fig. 5:**
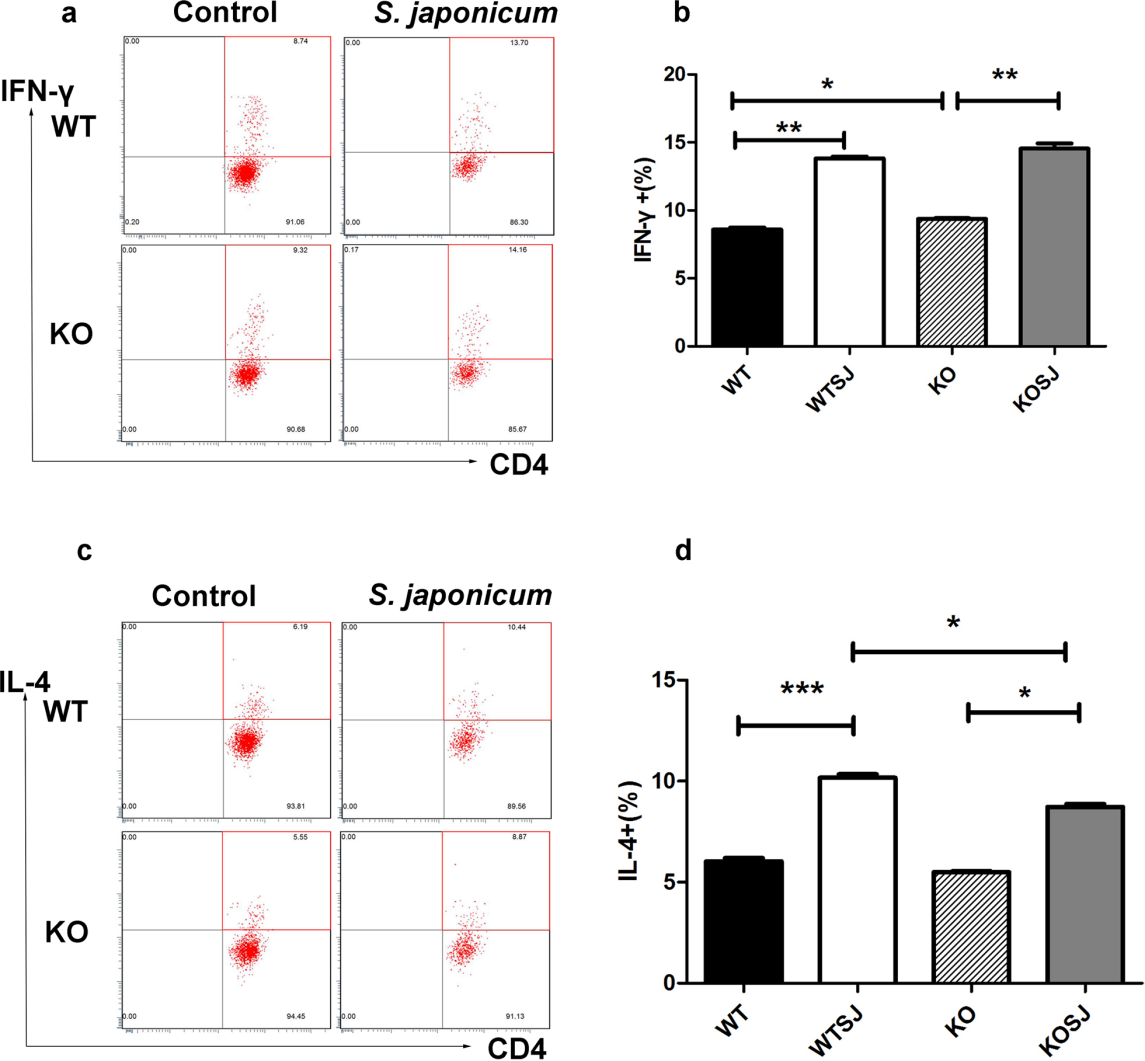
Flow cytometric analysis of CD4^+^ T cells in the spleen of infected mice. **a** Scatter diagram of Th1 cells. **b** Statistical analysis of the percentage of Th1 cells in each group. **c** Scatter diagram of Th2 cells. **d** Statistical analysis of the proportion of Th2 cells in each group (*P < 0.05; **P < 0.01; ***P < 0.001)

The intracellular cytokine profile of CD4^+^T in the spleen was determined by performing flow cytometry analysis. The proportions of CD4^+^IFN-γ^+^ cells in WT group, WTSJ group, KO group and KOSJ group were 8.60 ± 0.15%, 13.80 ± 0.13%, 9.40 ± 0.05% and 14.55 ± 0.38%, respectively (Fig. 5a, b). Our data indicate that schistosome infection could induce the increase of Th1 cell proportion in spleen; nevertheless, no statistical significance between WTSJ and KOSJ could be determined (*P* > 0.05). The levels of CD4^+^IL-4^+^ T cells in WT group, WTSJ group, KO group and KOSJ group were 6.00 ± 0.17%, 10.20 ± 0.17%, 5.50 ± 0.06% and 8.70 ± 0.14%, respectively (Fig. 5c, d). Hence, we observed that the Th2-skewed immune response induced by schistosome infection was significantly diminished because of the effect of PKCλ/ι knockout, as indicated by notably reduced Th2 cell proportions. These above results concur with the mRNA level of cytokines in the liver, collectively demonstrating that PKCλ/ι knockout could prevent the polarization of Th2-type immune response induced by schistosome infection.

## Discussion

In the course of *S. japonicum* infection, the repair of space-occupying lesions and immune stimulation of egg antigen jointly induce Th2-type immune response in the host [13, 14]. Given that the predominant Th2-type immune response restrains Th1-type inflammatory response, Th1/Th2 functional imbalance coinciding with the progression of the disease will damage liver tissue. The immune system modulates the biological functions of the immune cells, such as cytotoxic T cells, NK cells, dendritic cells and macrophages, via secretion of cytokines, causing the involvement of cell-cell interactions through the corresponding surface molecules [15]. Cytokines, such as IL-4 and IL-13, which are secreted by Th2 cells, can activate hepatic stellate cells and secrete a large amount of matrix and collagen fibers, rendering the liver more susceptible to damage. The excessive repair of the lesions tends to deteriorate the pathological fibrosis [16–19].

In the present study, we analyze the effect of conditional knockout of PKCλ/ι on liver fibrosis induced by *S. japonicum* and its influence on the development of the worms. The burdens of *S. japonicum* adults and eggs in the livers of KOSJ group were not notably different in contrast to WTSJ group, indicating that PKCλ/ι knockout could not affect the development and reproductive capacity of *S. japonicum*. H&E and Masson staining show that the degree of granulomatous lesions and fibrosis in the liver of the infected PKCλ/ι-knockout mice was significantly reduced compared with that of the infected wild-type mice (Fig. 3b, c, *P* < 0.001). The results of RT-PCR showed significantly lower mRNA expression levels of Col-1 and Col-3 in the liver tissues of KO than WT group. The immunoblotting of Col-1 and α-SMA in KOSJ mice was significantly less than that in WTSJ mice. Serological tests showed that the increasing levels of ALT in KOSJ group were obviously lower than those in WTSJ group. Consistently, our data confirmed that PKCλ/ι knockout can significantly alleviate the damage to liver tissue and impede the progression of fibrosis in mice caused by schistosome infection.

The pathological changes of liver fibrosis caused by schistosome could be determined by the liver cytokine microenvironment [20]. Th2 cytokines jointly play a pivotal role in the onset and progression of liver fibrosis caused by hepatosplenic schistosomiasis [21], while Th1-specific cytokine INF-γ is involved in the alleviation of hepatic fibrosis [22]. This could mostly be attributable to the molecular mechanism in which IL-4 is secreted by Th2 cells to tip the macrophage M1/M2 balance to M2 activation [23, 24]. Subsequently, M2-type macrophages secrete large amounts of IL-4 to increase Th2 polarization even further. TGF-β1 and other cytokines are secreted to activate the HSC to produce excessive collagen fibers, thereby promoting liver fibrosis [20]. RT-PCR showed no statistically significant difference in IFN-γ expression level between WTSJ group and KOSJ group. In contrast, the level of IL-4 in the liver of KOSJ group is significantly lower than that of WTSJ group, indicating that the liver microenvironment of Th2 polarization in PKCλ/ι-loss mice is diminished after schistosome infection. Considering the roughly plateaued IFN-γ-secreting Th1 cells, the Th1/Th2 paradigm was altered in the direction of the Th2-predominant immune response. This Th2 polarization is mostly attributed to schistosome egg antigens and their induced “cytokines field” [25]. The result of flow cytometry showed that schistosome infection led to increased count of IFN-γ secreted by Th1 CD4^+^ T cells in the spleens of WTSJ group and KOSJ group, yet the difference was not statistically significant. Conversely, the increasing extent of IL-4 secreted by Th2 CD4^+^T cells in KOSJ group was significantly lower than that of WTSJ group. The results showed that Th2 immune response in PKCλ/ι-deficient mice is increased after *S. japonicum* infection, but the rate of increase was lower than that in wild-type mice infected by *S. japonicum*. The secretion of Th2 cytokine requires continuous activation of TCR signaling. Jun-qi Yang et al. demonstrated that PKCλ/ι deficiency led to lowered IL-4 secretion and reduced phosphorylation activation of effector factor Stat6 in OVA-induced airway inflammation, thereby inhibiting the expression of nuclear transcription factor GATA3 in vitro [7]. In addition, PKCλ/ι deficiency can impair the NF-κB signaling pathway in Jurkat T cells. The inhibition of NF-κB activity can downregulate GATA3 expression and the production of Th2 cytokines [26]. Our results are consistent with those of Junqi Yang et al. [7] showing that PKCλ/ι deficiency may block the polarization of CD4 + T cells toward Th2, thereby reducing the degree of liver fibrosis caused by *S. japonicum*.

Excessive cellular apoptosis has been demonstrated in the host with schistosome infection and is probably attributed to the production of toxic factors by the residing schistosome eggs [27]. The cytokines released by immune cells, including macrophages, also participate in the inflammatory response of egg granuloma [28, 29]. Without the prompt intervention of phagocytes, the dead cells will release DAMPs (damage associated molecular patterns, DAMPs), such as HMGB1, into the surrounding micro-environment, exerting a harmful effect on the host [30]. Evidence indicates that HMGB1 released by dead cells could exacerbate liver fibrosis caused by schistosome infection via modulating HMGB1-TLR2/4-NF-κB signaling pathways or activation of HSC autophagy [31, 32]. The increased HMGB1 level in liver tissue of KOSJ group being significantly lower than that of WTSJ mice further demonstrates that PKCλ/ι knockout effectively reduces the percentage of dead liver cells and thereby lessens the degree of hepatic fibrosis.

To our knowledge, this study is the first that sheds light on the biological function of PKCλ/ι in reducing granulomatous and alleviating fibrotic lesions caused by schistosome infection in mouse via inhibiting Th2 polarization, which reaffirms the important role of PKCλ/ι in Th2 immune response.

## Conclusions

This research showed that PKCλ/ι knockout can effectively reduce the Th2 polarization of host immune response during *S. japonicum*, resulting in downregulation of IL-4, HMGB1 and TGF-β1, Col-1, Col-3 and α-SMA and can effectively alleviate hepatic egg granuloma and fibrosis induced by *S. japonicum* infection. The results of this study provide the scientific basis for the development of preventive and therapeutic measures for liver fibrosis by targeting PKCλ/ι.

## Data Availability

The datasets generated during the current study are included within the article.

## Abbreviations

Th2: T helper cells 2
*S. japonicum*: *Schistosoma japonicum*
RT-PCR: Real-time fluorescence quantitative polymerase chain reaction
ALT: Alanine aminotransferase
IL: Interleukin
TGF-β1: Transforming growth factor beta 1
Col-1: Collagen type I
Col-3: Collagen type III
DAMP: Damage-associated molecular patterns
HMGB1: High mobility group protein B1
PKC: Protein kinase C
OVA: Ovalbumin
HSC: Hepatic stellate cells
WT: Wild type
WTSJ: WT group mice infected with *S. japonicum*
KO: Knockout
KOSJ: KO group mice infected with *S. japonicum*
HYP: Hydroxyproline
IFN-γ: Interferon-γ
SEM: Standard error of mean
TNF-α: Tumor necrosis factor-α
PMA: Phorbol 12-myristate 13-acetate

## References

[1] Gryseels B, Polman K, Clerinx J, Kestens L (2006). Human schistosomiasis. Lancet.

[2] Qiu S, Fan X, Yang Y, Dong P, Zhou W, Xu Y (2017). *Schistosoma japonicum* infection downregulates house dust mite-induced allergic airway inflammation in mice. PloS ONE.

[3] Zhou W, Yang Y, Mei C, Dong P, Mu S, Wu H (2019). Inhibition of Rho-kinase downregulates Th17 cells and ameliorates hepatic fibrosis by *Schistosoma japonicum* infection. Cells.

[4] Carson JP, Ramm GA, Robinson MW, McManus DP, Gobert GN (2018). Schistosome-induced fibrotic disease: the role of hepatic stellate cells. Trends Parasitol.

[5] Colley DG, Bustinduy AL, Secor WE, King CH (2014). Human schistosomiasis. Lancet.

[6] Moscat J, Rennert P, Diaz-Meco MT (2006). PKCzeta at the crossroad of NF-kappaB and Jak1/Stat6 signaling pathways. Cell Death Differ.

[7] Yang JQ, Leitges M, Duran A, Diaz-Meco MT, Moscat J (2009). Loss of PKC lambda/iota impairs Th2 establishment and allergic airway inflammation in vivo. Proc Natl Acad Sci USA.

[8] Wynn TA (2004). Fibrotic disease and the T(H)1/T(H)2 paradigm. Nat Rev Immunol.

[9] Xhangolli I, Dura B, Lee G, Kim D, Xiao Y, Fan R (2019). Single-cell analysis of CAR-T cell activation reveals a mixed Th1/Th2 response independent of differentiation. Genom Proteom Bioinf.

[10] Wynn TA (2011). Fibrosis is regulated by Th2 and Th17 responses and by dynamic interactions between fibroblasts and macrophages. Am J Physiol Gastrointest Liver Physiol.

[11] Yang Y, Dong P, Zhao J, Zhou W, Zhou Y, Xu Y (2018). PKClambda/iota regulates Th17 differentiation and house dust mite-induced allergic airway inflammation. Biochim Biophys Acta Mol Basis Dis.

[12] Song LJ, Yin XR, Mu SS, Li JH, Gao H, Zhang Y (2020). The differential and dynamic progression of hepatic inflammation and immune responses during liver fibrosis induced by *Schistosoma japonicum* or Carbon Tetrachloride in mice. Front Immunol.

[13] Gieseck RL, Wilson MS, Wynn TA (2018). Type 2 immunity in tissue repair and fibrosis. Nat Rev Immunol.

[14] Pearce EJ, MacDonald AS (2002). The immunobiology of schistosomiasis. Nat Rev Immunol.

[15] Spangler JB, Moraga I, Mendoza JL, Garcia KC (2015). Insights into cytokine-receptor interactions from cytokine engineering. Annu Rev Immunol.

[16] Gordon S (2003). Alternative activation of macrophages. Nat Rev Immunol.

[17] Minutti CM, Knipper JA, Allen JE, Zaiss DM (2017). Tissue-specific contribution of macrophages to wound healing. Semin Cell Dev Biol.

[18] Van Dyken SJ, Locksley RM (2013). Interleukin-4- and interleukin-13-mediated alternatively activated macrophages: roles in homeostasis and disease. Annu Rev Immunol.

[19] Wynn TA, Vannella KM (2016). Macrophages in tissue repair, regeneration, and fibrosis. Immunity.

[20] Kamdem SD, Moyou-Somo R, Brombacher F, Nono JK (2018). Host regulators of liver fibrosis during human schistosomiasis. Front Immunol.

[21] Nono JK, Ndlovu H, Aziz NA, Mpotje T, Hlaka L, Brombacher F (2017). Host regulation of liver fibroproliferative pathology during experimental schistosomiasis via interleukin-4 receptor alpha. Plos Neglect Trop D.

[22] Henri S, Chevillard C, Mergani A, Paris P, Gaudart J, Camilla C (2002). Cytokine regulation of periportal fibrosis in humans infected with *Schistosoma mansoni*: IFN-gamma is associated with protection against fibrosis and TNF-alpha with aggravation of disease. J Immunol.

[23] Liu B, Jiang J, Liang H, Xiao P, Lai X, Nie J (2021). Natural killer T cell/IL-4 signaling promotes bone marrow-derived fibroblast activation and M2 macrophage-to-myofibroblast transition in renal fibrosis. Int J Immunopharmaco.

[24] Fu C, Jiang L, Hao S, Liu Z, Ding S, Zhang W (2019). Activation of the IL-4/STAT6 signaling pathway promotes lung cancer progression by increasing M2 myeloid cells. Front Immunol.

[25] Haeberlein S, Obieglo K, Ozir-Fazalalikhan A, Chaye MAM, Veninga H, van der Vlugt L (2017). Schistosome egg antigens, including the glycoprotein IPSE/alpha-1, trigger the development of regulatory B cells. PLoS Pathog.

[26] Das J, Chen CH, Yang L, Cohn L, Ray P, Ray A (2001). A critical role for NF-kappa B in GATA3 expression and TH2 differentiation in allergic airway inflammation. Nat Immunol.

[27] Lundy SK, Lerman SP, Boros DL (2001). Soluble egg antigen-stimulated T helper lymphocyte apoptosis and evidence for cell death mediated by FasL(+) T and B cells during murine *Schistosoma mansoni* infection. Infect Immun.

[28] Doenhoff MJ, Pearson S, Dunne DW, Bickle Q, Lucas S, Bain J (1981). Immunological control of hepatotoxicity and parasite egg excretion in *Schistosoma mansoni* infections: stage specificity of the reactivity of immune serum in T-cell deprived mice. T Roy Soc Trop Med H.

[29] Tilg H (2001). Cytokines and liver diseases. Can J Gastroenterol.

[30] Vicentino ARR, Carneiro VC, Allonso D, Guilherme RF, Benjamim CF, Dos Santos HAM (2018). Emerging role of HMGB1 in the pathogenesis of schistosomiasis liver fibrosis. Front Immunol.

[31] Li J, Zeng C, Zheng B, Liu C, Tang M, Jiang Y (2018). HMGB1-induced autophagy facilitates hepatic stellate cells activation: a new pathway in liver fibrosis. Clin Sci.

[32] Li X, Jin Q, Yao Q, Xu B, Li Z, Tu C (2016). Quercetin attenuates the activation of hepatic stellate cells and liver fibrosis in mice through modulation of HMGB1-TLR2/4-NF-κB signaling pathways. Toxicol Lett.

